# Celiac crisis as the life-threatening onset of celiac disease in children: a case report

**DOI:** 10.3389/fped.2023.1163765

**Published:** 2023-05-12

**Authors:** Angela Mauro, Francesca Casini, Antonella Talenti, Clelia Di Mari, Anna Rita Benincaso, Giovanni Di Nardo, Luca Bernardo

**Affiliations:** ^1^ Department of Childhood and Developmental Medicine, Fatebenefratelli Hospital, Milan, Italy; ^2^ Department of Biomedical and Clinical Sciences, Buzzi Children's Hospital, University of Milan, Milan, Italy; ^3^ Faculty of Medicine and Psychology, Sapienza University of Rome-NESMOS Department, Sant’Andrea University Hospital, Rome, Italy

**Keywords:** celiac crisis, celiac disease, pediatrics, hypoalbuminemia, diarrhea, case report

## Abstract

Celiac disease (CD) is an immune-mediated enteropathy caused by a permanent sensitivity to gluten in genetically susceptible individuals. In rare cases, CD may occur with a severe potential life-threatening manifestation known as a celiac crisis (CC). This may be a consequence of a delayed diagnosis and expose patients to possible fatal complications. We report the case of a 22-month-old child admitted to our hospital for a CC characterized by weight loss, vomiting, and diarrhea associated with a malnutrition state. Early identification of symptoms of CC is essential to provide a prompt diagnosis and management.

## Introduction

Celiac disease (CD) is a systemic and chronic immune-mediated disease that occurs in genetically predisposed individuals after dietary exposure to gluten (1). It is characterized by the presence of celiac-specific autoantibodies and inflammation of the small intestine, associated with a wide spectrum of gastrointestinal and extraintestinal symptoms, resembling a multisystemic disorder (2).

CD prevalence is around 1% in most populations, with an increasing trend in the last few years due to both a rise in knowledge and increased autoimmunity, as revealed by seroprevalence reports of apparently asymptomatic subjects (3–6). The only effective treatment of CD is a life-long gluten-free diet (GFD) (7). Being a highly prevalent worldwide condition, CD has an important burden on lifestyle, but its evaluation is limited by undiagnosed asymptomatic patients and the lack of pharmacologic treatment (1).

Economic analyses demonstrated high costs, especially at the diagnosis, related to investigations and monitoring of the disease (8, 9). Moreover, the non-economic impact of the diet must be considered due to its relevance to psychological and social well-being (10, 11). The clinical presentation of CD is heterogeneous. Gastrointestinal symptoms, including constipation, bloating, vomiting, diarrhea, and recurrent abdominal pain, are the most frequent symptoms in the pediatric age range; however, extraintestinal or atypical symptoms such as dermatitis, failure to thrive, headache, anemia, delayed puberty, or dental enamel defects may be signs of CD (12–14).

Celiac crisis (CC) is a severe medical emergency characterized by an acute onset or rapid progression of gastrointestinal symptoms requiring hospitalization and/or parenteral nutrition along with profuse diarrhea and consequent severe dehydration, electrolyte imbalance, and hypoalbuminemia associated with neurologic and renal dysfunction.

The diagnostic criteria of CC are shown in detail in Table 1 (15–17).

**Table 1:** Diagnostic criteria of celiac disease.

Acute onset or rapid progression of gastrointestinal symptoms attributable to celiac disease with almost two of the following criteria:
• Severe dehydration including orthostatic changes • Renal dysfunction • Neurological dysfunction • Metabolic acidosis • Abnormal electrolyte levels, including hyponatremia, hypokalemia, hypocalcemia, or hypomagnesemia • Hypoproteinemia (albumin level <3 g/dL) • Weight loss, >4.5 kg

CC is usually regarded as a complication of previously undetected celiac disease, rarely a consequence of non-compliance with the recommended GFD (16, 17).

We describe a case of a patient admitted to our emergency department for a severe life-threatening celiac crisis as the first manifestation of a previously unknown CD.

## Case presentation

A 22-month-old boy was admitted to our emergency department because of episodes of vomiting after meals, associated with appetite loss and watery diarrhea, without fever. Symptoms have been noticed for the last 2 months. Parents also reported significant weight loss over the previous 6 months.

The patient was born at 37 weeks of gestation, had no significant perinatal history, and was breastfed for the first 9 months. No relevant clinical history was reported. All mandatory vaccinations were performed. His family history was unremarkable.

At our first evaluation, physical examination showed a dystrophic appearance with scarce subcutaneous tissues and signs of moderate dehydration (increased capillary refill time, sticky mucous membranes). Abdominal bloating and distension were present without any pain from palpation. The cardiothoracic examination was normal. Vital parameters were within range. His weight was 11.930 kg (0 SD according to WHO charts), while his length was 94 cm (between +2 and +3 SD according to WHO charts), with a body mass index of 13.50 kg/m^2^ (between −3 and −2 SD according to WHO charts).

The patient was admitted to our pediatric department for adequate investigation and treatment. Laboratory tests revealed normal full blood counts (leucocytes 14,780/MMC: N 44.7%, L 48.2%; Hb 14.7 g/dL, platelets 396,000/MMC) and electrolytes, negative inflammatory markers, and normal coagulation but a state of malnutrition (serum albumin level 2.56 g/dL, serum ferritin 9.6 ng/mL, vitamin D-25-OH 16.1 ng/mL, folate 1.99 ng/mL) and a slight increase of the transaminase levels (AST 73 U/L, ALT 62 U/L). Serology assays for HIV, CMV, HCV, HBV, and HAV were negative. Antinuclear antibodies were negative. The urine test was negative for proteinuria. Stool cultures for *Salmonella*, *Shigella*, *Campylobacter*, *Clostridium difficile*, *Giardia* sp., *Cryptosporidium* sp., *Entamoeba histolytica*, and fecal antigens for rotavirus and adenovirus were negative. The abdomen ultrasound was normal. Thyroid function analysis revealed hypothyroidism (TSH 7.92 microUI/mL, FT4 0.78 ng/mL); antithyroid peroxidase antibodies were 256.8 IU/mL (vn < 35 UI/mL) with normal value of antithyroglobulin antibody, and thyroid echography showed features of thyroiditis. Specific CD serological tests showed the presence of IgA anti-TG2 328 U/mL (n.v < 7 U/mL); IgA antiendomysial antibodies (EMA) were positive. Genetic testing revealed the presence of HLA DQA1*05,0201 and DQB1*0302.

Considering IgA anti-TG >10 times the normal value and EMA positivity in a second blood sample, the patient was diagnosed with CD according to the recent European Society for Pediatric Gastroenterology, Hepatology and Nutrition (ESPGHAN) guidelines, without undergoing esophago-gastro-duodenoscopy (7).

An intravenous infusion of albumin (0.5 g/kg) and furosemide (1 mg/kg) was performed to correct hypoalbuminemia. Total parenteral nutrition was started and continued for 11 days. Intravenous methylprednisolone (1 mg/kg/day), progressively tapered, was administered for 10 days. Contextually, folate, vitamin D, and vitamin B12 were supplemented, and replacement thyroid therapy was started with levotiroxina 25 mg/day. Once clinically stable, a GFD was started.

During hospitalization, clinical conditions, hydration, and nutritional status slowly improved. Abdomen bloating resolved; stools became normal.

He was discharged on day 22 of hospitalization with a weight of 12 kg (0 SD according to WHO charts). Vitamin supplementation and thyroid hormone therapy were continued.

**Figure 1:**
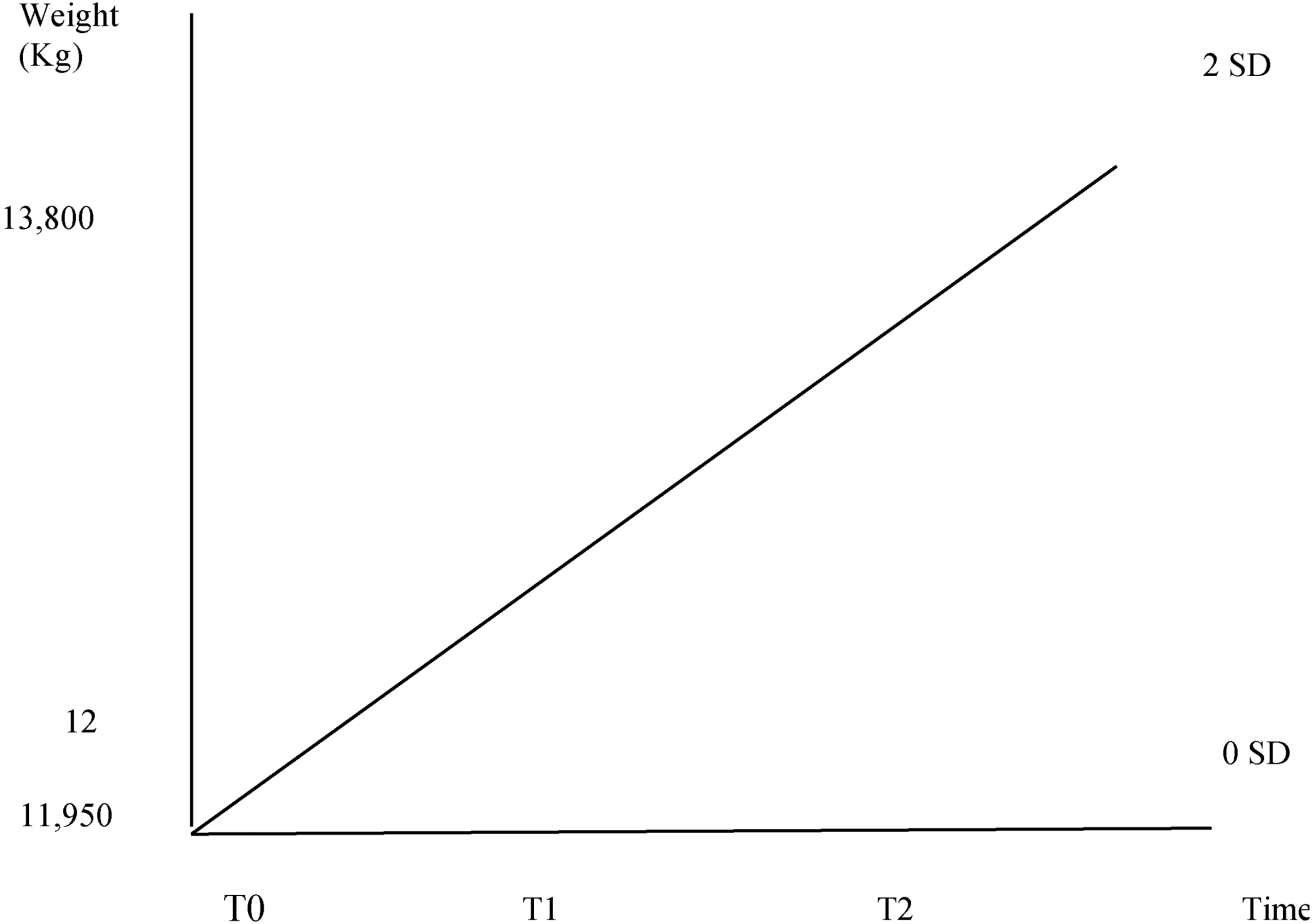
Progressive weight increase at T0 (admission)—T1 (discharge)—T2 (1-month follow-up).

At 1 month's follow-up visit, he showed optimal general conditions and no nutritional deficits. His weight was 13.800 kg (between 0 and +2 SD according to WHO charts) (Figure 1).

Informed consent was received from the parents for the publication of this case report.

## Discussion

CC is an urgent and life-threatening complication of CD (2, 18–21). The incidence of CC is not exactly known. Babar et al. reported a 5% incidence of CC (14); however, it has been considered a rarer manifestation of CD in other studies (22, 23). CC was first described by Andersen and Di Sant’agnese in 1953 when he reported the cases of 35 patients with CC, among which three were complicated by fatality (24)_._ The mean age at presentation is generally early childhood, with highly variable ranges depending on the geographic area: in developing countries, CC onset is at a mean age of 5 years, while in high-outcome countries, such as our case report, a higher incidence of CC occurs in the first 2 years of life (25). Nevertheless, its frequency has drastically decreased in the last decades, probably due to a higher rate of early diagnosis. Moreover, vaccination, infection control, management, and availability of gluten-free diet have decreased the risk of CC (17, 25, 26).

CC may be a complication of an unrecognized CD or, less commonly, a consequence of non-compliance with a previously recommended GFD. CC must be differentiated from non-responsive-CD which is a type of CD which does not respond after 6–12 months on a GFD, extremely rare in children (27).

CC is characterized by severe acute onset of gastrointestinal symptoms associated with almost two of the diagnostic criteria reported in Table 1 (16, 17, 26). Without appropriate intervention, clinical conditions may rapidly worsen with electrocardiographic abnormalities, neuromuscular weakness, tetany, seizures, acute kidney injury, and circulatory collapse (28–30). CC may also be associated with other rare symptoms, such as neurological manifestations, including ataxia and myoclonus, and hematological manifestations, such as thrombocytopenia (14, 31).

Even if not specific to CD, these symptoms can hint at the diagnosis of CC if a CD is already diagnosed; in the case of previously undetected CD, a differential diagnosis with other conditions could be considered (Table 2).

**Table 2:** Differential diagnosis of celiac crisis.

Infectious disease	Parasitic infestation Hp-positive gastritis and peptic duodenitis Tropical sprue Bacterial overgrowth Wipple disease Viral gastroenteritis or postinfectious changes
Drugs	Non steroidal anti-inflammatory drugs Antineoplastic and immune modulatory drugs (including immune checkpoint inhibitors) Angiotensin receptor blockers use (olmesartan and others)
Other immune-inflammatory conditions	Collagenous sprue Immunodeficiencies (including common variable immunodeficiency) Autoimmune enteropathy Crohn's disease and ulcerative colitis-associated duodenitis Eosinophilic gastroenteritis and food protein-sensitive enteropathies (including gluten-sensitive enteropathy)

Due to its severe presentation, the possible alternative diagnosis could be infectious diarrhea. Therefore, a history of potential microbial exposures and laboratory data should be obtained to rule out infectious etiologies. Other conditions to consider that may mimic CC are tropical sprue, AIDS enteropathy, hypersensitivity to dietary proteins, Wipple's disease, intestinal lymphoma, collagenous sprue, small intestinal bacterial overgrowth, use of laxatives, and pancreatic insufficiency.

In any case, patients with acute, severe, rapidly progressive gastrointestinal symptoms, if an infection is ruled out, should be tested for CD (15–17).

Therapeutic management of CC mainly consists of dealing with the life-threatening consequences of profuse diarrhea by fluid resuscitation, intravenous correction of the electrolyte imbalance, and albumin correction (27–32). Parenteral nutrition is generally required considering the massive malabsorption syndrome. As soon as clinically possible, starting a GFD represents the only etiologic treatment (33).

Corticosteroid's role in CC is controversial. In the literature, a good response of CC to steroids has been generally reported, as in our case report, since they can reduce intestinal inflammation and restore the brush border enzymes (34–37); however, Gupta and Kapoor reported two cases who deteriorated on steroids and one who improved without steroids: attention should be paid in their use mainly in the case of hypokalemia, which can worsen under steroids' kaliuresis effect, and in the case of probable underlying sepsis (38).

Our patient presented acute gastrointestinal symptoms associated with three of the diagnostic criteria (Table 1). The diagnosis of CD was based on the value of IgA anti-TG (>10 times the normal value) and EMA positivity, according to the recent ESPGHAN guidelines (7).

Moreover, as in our case report, an association between CD and autoimmune thyropathy (AIT) has been described (39). According to prevalence studies, the association between AIT and CD ranges from 2.4% to 40.4%, although their relationship is controversial (40–44). It was supposed that CD and AIT share one or more susceptibility genes, and the prolonged consumption of gluten in patients with undiagnosed CD may damage the intestinal barrier, leading to an alteration of the immune system (40). Other studies, instead, reported that exposure to gluten does not influence the development or the progression of autoimmune diseases (such as AIT, insulin-dependent diabetes mellitus, Addison disease, and primary Sjogren's syndrome) and GFD does not lessen the progression of autoimmune disease (45, 46). Guariso et al. assessed that GFD seems to have a good impact on autoimmune disease, although it does not have any effect on the progression of the autoimmune process if already started (47).

## Conclusion

The present case highlights the possibility of CC as the first manifestation of CD. Although rarely encountered in clinical practice, this abrupt onset of CD requires hospitalization and immediate treatment (i.e., protein replacement, vitamin supplementation, and GFD) to avoid life-threatening complications. Therefore, prompt identification of symptoms has a crucial role in early diagnosis, management, risk stratification, and association with other autoimmune diseases.

## Data Availability

The original contributions presented in the study are included in the article, further inquiries can be directed to the corresponding author.
